# A Study on the Synthesis and Proton Transport Behavior of Multilayered ZSM-5 Zeolite Nanosheet Membranes Laminated on Polymer Substrates

**DOI:** 10.3390/membranes13030305

**Published:** 2023-03-06

**Authors:** Zishu Cao, Landysh Iskhakova, Xinhui Sun, Junhang Dong

**Affiliations:** Department of Chemical and Environmental Engineering, University of Cincinnati, Cincinnati, OH 45221, USA

**Keywords:** zeolite nanosheet, polymer, laminated membrane, proton conduction

## Abstract

Single crystalline ZSM-5 ZNs with thicknesses around 6 nm were obtained by secondary growth of silicalite nanoparticles using diquaternary bis-1,5(tripropyl ammonium) pentamethylene diiodide (dC_5_) as a structure-directing agent (SDA). The dC_5_ could be effectively removed from the ZN pores by either high-temperature calcination or UV irradiation in air at room temperature but not by the piranha solution treatment. Ultrathin ZN-laminated membranes (ZNLMs) were fabricated by sandwiching a UV-activated multilayered ZN film between two recast Nafion^®^ layers (ZNLM-Nafion) and by filtration coating from a suspension of thermally activated ZNs on a nonionic porous PVDF (ZNLM-PVDF). The ZNLMs on both supports demonstrated the ability of highly proton-selective ion conduction with low resistances in aqueous electrolyte solutions. The ZNLM-PVDF with PVDF binder was structurally stable, and it achieved a comparably low ASR but much higher proton selectivity compared with a Nafion membrane of same overall thickness. However, detachment between the ZNLM and Nafion layers occurred when the ZNLM-Nafion operated in aqueous electrolyte solutions. Results of this study show the potential for developing ZNLMs as efficient proton-conducting membranes without using expensive ionic polymer matrices. However, the development of polymer-supported ZNLMs is hindered by the current inefficiency in preparing well-dispersed suspensions of open-pore ZNs. Future development of efficient methods for synthesizing open-pore ZNs in dispersed states is key to realizing high-performance ZNLMs on polymers.

## 1. Introduction

**Figure 1:**
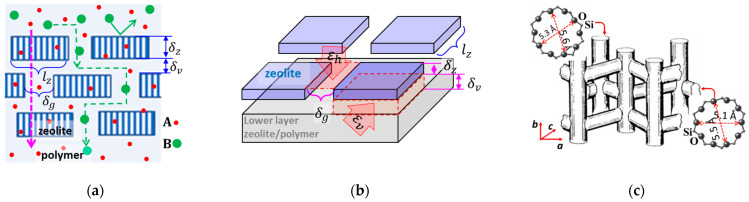
Schematics showing (**a**) the cross-section structure of a composite membrane with zeolite crystals embedded in a polymer matrix, (**b**) the molecular transport pathways in a membrane containing zeolites that are permeable to molecule “A” but impermeable to “B”, and (**c**) the topological structure of the interconnected 3-dimensional channel system in the MFI-type zeolites.

Molecular sieve zeolite crystals can be embedded in polymer membranes to enhance selectivity and permeability in molecular and ionic separations [1]. As schematically shown in Figure 1a,b, the embedded discrete zeolite crystals allow penetration for molecules smaller than the zeolitic pore size while rejecting those larger than the pore size. The larger molecules or ions rejected by the zeolitic pores can penetrate through the membrane only by diffusing along the tortuous inter-crystal pathways at a reduced rate that improves the transport selectivity towards the smaller molecules. The selectivity of the composite membranes depends on the spaces between the embedded zeolite crystals and the individual crystal area and aspect ratio $R_{Z}=l_{z}/\delta_{z}$, (where $l_{z}$ and $\delta_{z}$ are characteristic side length and thickness of a slab-shaped crystal, respectively). The selectivity of the composite membrane can be enhanced by reducing the inter-crystal spaces (i.e., $\delta_{v}$ and/or $\delta_{h}$) and increasing $R_{Z}$ when the zeolitic channels are oriented along the thickness. Figure 1c illustrates the 3-dimensional channel system in the MFI-type zeolite, which comprises near-cylindrical straight channels along the *b*-axis interconnected with sinusoidal channels running in the a–c plane [2]. Because of the slightly bigger opening (dia. ~0.56 nm) and shorter length of the straight channels, an MFI zeolite membrane with thickness oriented in the *b*-axis direction thus provides higher fluxes than those with other crystal orientations.

The 2-dimensional (2D) zeolite nanosheets (ZNs) with large $R_{z}$ and nanometre $\delta_{z}$ in the *b*-axis direction offer the opportunity to construct zeolite-polymer composite membranes with high selectivity and low transport resistances. As depicted by Figure 1b, for a membrane with monolayer ZNs, the separation selectivity depends on the width ($\delta_{h}$) and porosity ($\varepsilon_{h}$) of horizontal inter-ZN gaps in the membrane surface. The molecular or ion sieving selectivity of such monolayer ZN membranes can be maximized by converting the discrete ZNs into an intergrown polycrystalline film via secondary growth that leads to $\delta_{h}\to 0$ and $\varepsilon_{h}\to 0$ [3].

However, the formation of an intergrown monolayer ZN membrane requires smooth inorganic substrates to avoid misalignments of the ZN layer and allow zeolite activation after the secondary growth that is impractical for polymer substrates. A ZN-based membrane on more affordable and amendable polymer supports without secondary growth for horizontal inter-ZN gap elimination is practically desirable. Recently, multilayered MFI-type ZN-laminated membranes (ZNLM) on porous polymer films have been demonstrated to offer high selectivity and fluxes in molecular separations [4,5]. As schematically shown in Figure 1a,b, in the multilayered ZNLMs, the $\delta_{h}$ is inevitably much larger than the $\delta_{v}$ between overlapping ZNs; hence, the membrane selectivity is mainly determined by the width ($\delta_{v}$) and porosity ($\varepsilon_{v}$) of inter-ZN spaces in thickness direction. The $\varepsilon_{v}$ may be estimated by

(1) $$\varepsilon_{v}=\frac{4\cdot l_{z}\cdot \delta_{v}}{l_{z}^{2}+l_{z}\cdot \delta_{h}}<\frac{4\cdot \delta_{v}}{l_{z}}$$

The polymer-supported ultrathin ZNLM layers without binders [4] or with minimal polymer binders between ZNs [5] could minimize $\delta_{v}$ to substantially improve the selectivity and flux compared with conventional composite membranes of polymers embedded with isotropic zeolite crystals. Recently, the MFI-type ZNs have been attracting growing interest for membrane development because of the potential to achieve high performances in many impactful petrochemical purification and ion separation processes permitted by its 10-membered ring medium pore size and extraordinary structural stability [6,7,8].

In the literature, single crystal MFI ZNs have been synthesized by a silicalite nanoparticle-seeded secondary growth method using the diquaternary bis-1,5(tripropyl ammonium) pentamethylene diiodide (dC_5_) as a structure-directing agent (SDA) [6,7]. The as-synthesized ZNs are impermeable because the zeolitic pores are occupied by the large SDA molecules. These SDA molecules are most effectively and conveniently removed by calcination in air at >450 °C. However, the high-temperature calcination process inevitably causes aggregation of the ZNs, which are difficult to breakup or redisperse. Thus, synthesis of ZNLMs on polymer substrates, which requires suspensions of well-dispersed open-pore ZNs, has been challenging with limited progresses reported so far.

To avoid the solid-state irreversible aggregation, Zhang et al. [4] activated pure-silica MFI (i.e., silicalite) ZNs ($l_{z}$~1.0 um, $\delta_{z}$~3.2 nm, and $R_{z}$~312) by multiple cycles of treatment in piranha solution (H_2_SO_4_:H_2_O_2_ volume ratio of 3:1) to decompose the C_22_H_45_–N^+^(CH_3_)_2_–C_6_H_12_–N^+^(CH_3_)_2_-C_6_H_13_](Br_2_) (C_22-6-6_) SDA. This liquid phase activation process provided well-dispersed open-pore ZN suspensions. The preactivated ZN suspension was used for coating binder-free ZNLMs on porous polybenzimidazole supports by vacuum filtration. The binder-free ZNLM had minimized $\delta_{v}$ to reduce the less selective inter-ZN transport, and that in turn achieved the molecular sieving effect between *n*-butane and *i*-butane. However, the binder-free multilayered ZNLM is likely to lack structural stability in liquid phase, especially in electrolyte solutions where the layered ZNs could disintegrate and redisperse upon surface solvation or ionization.

In a previous work, we synthesized aluminum-containing MFI (i.e., ZSM-5) ZNLM on porous polyvinylidene fluoride (PVDF) support using a minimal amount of PVDF as binder to prevent the multilayered ZNs from dissociation in concentrated slat solutions [5]. The suspension of open-pore ZSM-5 ZNs was obtained after calcination by multiple cycles of sonicated dispersion and sedimentation and centrifugation to remove the ZN agglomerates. The individual ZSM-5 ZNs had $l_{z}$~0.85 um, $\delta_{z}$~7.0–7.5 nm, and $R_{z}$~113. The ZNLM exhibited nearly perfect slat rejection (>99%) and high water flux (>6 L/h‧m^2^) during pervaporation desalination of brines with total dissolved salts of ~22 wt.%. The efficient ion rejection was achieved because the nanometer-scale $\delta_{v}$ effectively limited the inter-ZN salt migration under fast water transport rates through the ZNs. However, this ZNLM was unable to exhibit the molecular sieving effect for *n*-/*i*-butane because its $\delta_{v}$ was enlarged by the inserted binder polymer chains compared with that of the binder-free ZNMLs [4].

More recently, the MFI-type ZNs were used to fabricate a composite membrane by embedding well-aligned ZNs in a Nafion^®^ matrix film [8]. This ZN-embedded Nafion membrane exhibited improved proton-to-vanadyl ion selectivity because of the ion sieving effect of the MFI pores without increasing the electric resistance due to the nanometer thickness of ZNs [8,9]. This ZN-Nafion mixed matrix membrane was demonstrated as an ion separator to significantly improve the efficiency of the vanadium redox flow battery (VRFB) compared with the pure Nafion membrane. However, the use of expensive ionic polymers, especially the environmentally problematic sulfonated tetrafluoroethylene-based polymer (e.g., Nafion^®^), is practically unfavorable. On the other hand, studies on the fabrication and ion conduction behavior for ZNLMs coated on ubiquitous nonionic polymer supports are so far very limited. In the present work, we investigate the MFI ZN activation by different methods for coating ultrathin ZNLMs on the nonionic macroporous PVDF and Nafion^®^ films, respectively. The synthesized ZNLMs are studied for proton-selective ion conduction in electrolyte solutions.

## 2. Experimental

### 2.1. Chemicals and Materials

The chemicals and materials used in this work included tetraethyl orthosilicate (TEOS, >99%, Sigma-Aldrich, St. Louis, MO, USA), tetrapropylammonium hydroxide (TPAOH, 1 M aqueous, Sigma-Aldrich, USA), sodium aluminate (50–56 wt.% as Al_2_O_3_, Sigma-Aldrich, St. Louis, USA), sulfuric acid (95–98%, Sigma-Aldrich, St. Louis, USA), vanadium(IV) sulfate oxide hydrate (99.9%, Alfa Aesar, Haverhill, MA, USA), magnesium sulfate (≥99.5%, Sigma-Aldrich), hydrogen peroxide (30%, Fisher Scientific, Waltham, MA, USA), Nafion ^®^ perfluorinated resin solution (20%, Sigma-Aldrich, St. Louis, USA), ethanol (>99.5%, Sigma-Aldrich, USA), methanol (>99.8%, Sigma-Aldrich, St. Louis, USA), N,N-dimethylformamide (DMF, 99.8%, Sigma-Aldrich, St. Louis, USA), dimethyl sulfoxide (DMSO, 99.8%, Sigma-Aldrich, St. Louis, USA), hydrophilic surface PDVF film (average pore diameter d_p_ of 0.22 µm and porosity $\varepsilon_{PVDF}$ of >80%, TISCH Scientific, Fremont, CA, USA), hydrophilic surface PVDF film (d_p_~0.45 µm and $\varepsilon_{PVDF}$~80%, TISCH Scientific, CA, USA), and deionized water.

### 2.2. Material Characterizations

The morphological properties of the zeolite materials, i.e., nanoparticles, ZNs, and thin films, were examined by scanning electron microscopy (SEM) and energy dispersive X-ray spectroscopy (EDS) using a FEI Scios DualBeam microscope equipped with Ametek Octane Super EDAX. The N_2_ adsorption and desorption BET analysis was performed at 77 K by a Micromeritics ASAP 2020 unit. The zeolite films activated by UV irradiation were also characterized by attenuated total reflection-Fourier transform infrared spectroscopy (ATR-FTIR, Thermo Scientific Nicolet iS50 FT-IR spectrometer) to verify the removal of organic SDA molecules from the zeolite channels.

### 2.3. ZSM-5 ZN Synthesis

The ZSM-5 ZNs were synthesized by the uninterrupted two-stage secondary growth process using the silicalite nanoparticle seeds and dC_5_ SDA. The dC_5_ was synthesized in-house via the reported method of exhaustive alkylation of 1,5-diaminopentane with 1-iodopropane [6,7]. The silicalite nanoparticle seeds were prepared by a procedure detailed in the same publications, which involved a two-step in situ crystallization process using a starting solution with a molar composition of 10 SiO_2_ + 2.4 TPAOH + 0.87 NaOH + 114 H_2_O [5]. This solution was obtained by mixing 0.16 g water, 0.127 g NaOH, 8.93 g of 1.0 M TPAOH aqueous solution, and 2.5 g silicic acid in a Teflon flask. The solution was stirred at room temperature overnight and then reacted in a closed Teflon autoclave at 50 °C for 6 days under static condition. After the 6-day reaction, the liquid phase of the mixture was collected by a GHP syringe filter (pore size 0.45 μm). The recovered clear solution was subsequently reacted in a Teflon autoclave at 100 °C for 3 days. The produced silicalite nanoparticles were recovered by centrifugation and washed with DI water until the suspension reached neutral pH (~7).

The synthesis of ZSM-5 ZNs was accomplished by the silicalite nanoparticle (SNP)-seeded secondary growth method described in our previous publications [5,7]. The hydrothermal reactions of silicalite nanoparticle-seeded ZN growth were conducted at 140 °C and autogenous pressure. The reactions were conducted in a custom-made Teflon-lined autoclave, which allowed injection of additional reactant to the closed autoclave without interrupting the reaction process [7]. The entire hydrothermal reaction had a duration of 4 days, including a first stage of 3.5-day reaction in an aluminum-free precursor with molar ratios of 80 TEOS: 3.75 dC_5_: 20 KOH: 9500 H_2_O and a continued second stage of 0.5-day reaction after injecting a controlled amount of 1 M NaAlO_2_ solution. Before the hydrothermal reaction, the aluminum-free precursor solution was aged at room temperature for 16 h under an Ar purging flow (50 mL/min) for reducing the ethanol content. The number of silicalite nanoparticle seeds added into the precursor was 1/200 of the TEOS on the basis of Si mass. The amount of injected NaAlO_2_ was calculated such that the Si/Al atomic ratio of the whole mixture reached 50.

After reaction, the autoclave was quenched to room temperature in tap water flow and the solid products were recovered by centrifugation and filtration. The solid products were immersed in a solution of 0.1 M KOH + 1 M KCl (pH~13) for 1 week to dissolve unreacted amorphous silica materials and then rinsed by DI water. The as-synthesized ZN crystals were treated with intensive sonication for 2 h in liquid water or ethanol (EtOH) filled with ϕ4 mm ZrO_2_ milling beads. These milling beads were used to mechanically breakup the originally rhombus-shaped ZN crystals of which each contained a seed-evolved thick core encircled by a flat ZN. The flat ZN flakes dissociated from the uniformly thin sheets encircling the center cores were then separated from the large debris of the core part by centrifugation. The mass of the separated flat ZNs was approximately 10% of the total rhombus crystals.

### 2.4. ZN Activation

The removal of dC_5_ SDA from the ZSM-5 ZNs was investigated by different techniques, including high-temperature calcination, UV irradiation in air, and piranha solution treatment. The UV irradiation method was demonstrated to be effective for decomposing the diquaternary [C_22_H_45_-N^+^(CH_3_)_2_-C_6_H_12_-N^+^(CH_3_)_2_-C_6_H_13_](Br_2_) (C_22-6-6_Br_2_) SDA in air to activate multilayered ZNs under dry conditions [10]. The piranha solution with an H_2_SO_4_/H_2_O_2_ volumetric ratio of 3:1 was also reported for oxidation removal of the diquaternary ammonium C_22-6-6_ SDA from silicalite ZNs that led to open-pore ZNs in a liquid-dispersed state [4].

For thermal activation, the dry ZNs were randomly spread out over the crucible bottom and then calcined in air at 500 °C for 6 h. The effectiveness of dC_5_ SDA removal by high-temperature calcination is confirmed by thermogravimetric analysis (TGA) in air flow. The thermally activated ZNs were then redispersed by intensive sonication for extended periods in liquid media of which the pH value was adjusted by HNO_3_ or KOH/NaOH solutions. The as-synthesized ZNs were also directly dried and fired without removing the amorphous residuals and subsequently dispersed in the 0.1 KOH + 1.0 KCl solution. This uncleaned sample was to test the use of amorphous silica existing between individual ZNs for assisting with redispersion of calcined ZNs. This was attempted based on the assumption that amorphous silica could be dissolved in the KOH solution to help dissociate the aggregated ZNs. All calcined ZNs were redispersed into liquid solvents to form suspension for ZNLM coating on polymers.

In the UV irradiation method, a thin ZN film was deposited on the hydrophobic PVDF film (d_p_~0.22 µm) from a suspension containing 0.02 wt.% ZNs in a solvent of 80 wt.% water and 20 wt.% ethanol. The film was obtained by vacuum filtration of 3 mL of suspension over a 9.1 cm^2^ disc-shaped porous PDVF film. The procedure of UV activation of the ZN film was similar to the literature-reported method for activating SSZ-13 membranes [11]. The ZN film was placed to face the UV lamp (Mineralight^®^ R-52G) at a distance of 3 mm from the UV bulb. The UV irradiation activation used a typical duration of 6 days. The removal of dC_5_ SDA was examined by EDS and FTIR analyses. These elemental analyses were performed for ZN film coated on an alumina disc (d_p_~0.1 μm) to avoid interferences of the targeted elements from the PVDF support. The ZN film on alumina was obtained by 10 s of dip-coating in a suspension containing 0.2 wt.% ZNs and activated by the UV irradiation process with varied duration.

The piranha solution treatment used the basic procedure reported by Zhang et al. [4], which was effective for oxidizing and removal of the diquaternary ammonium SDA (C_22_H_45_–N^+^(CH_3_)_2_–C_6_H_12_–N^+^(CH_3_)_2_-C_6_H_13_)(Br_2_) from the nonionic silicalite ZNs. The treatment process included the following operations: (i) 0.1 g of dry zeolite nanosheets was first mixed with 6 mL of H_2_SO_4_ (95–98%) solution followed with a 5 min sonication treatment; (ii) 2 mL of hydrogen peroxide (H_2_O_2_, 30%) was added to the suspension and subsequently allowed to react at 80 °C overnight with the reactor mouth loosely covered; (iii) the ZNs were separated from the suspension by centrifugation after overnight reaction; and (iv) the ZNs were redispersed into the piranha solution and the steps mentioned above were repeated 4 times. The thus-treated ZNs were then separated and rinsed with DI water and dried for characterizations. The piranha solution activation process mentioned above was also conducted for wet ZNs separated directly after the hydrothermal synthesis. The piranha solution treatment of the wet ZN samples was to test the possibility of improving zeolitic pore accessibility to the oxidizing H_2_O_2_ by avoiding ZN aggregation in dry states. The results, however, showed no differences from the treatment of dry ZNs.

### 2.5. Fabrication of ZNLMs on Polymers

Two types of ZN-polymer composite membranes were fabricated for evaluating proton-selective ion conduction in aqueous solutions. These included a ZNLM formed by sandwiching a UV-activated continuous ZN film between two Nafion^®^ layers, denoted as ZNLM-Nafion hereafter, and a Nafion-free ZNLM coated on a macropore (d_p_~0.45 μm) hydrophilic PVDF film, denoted as ZNLM-PVDF hereafter. The ZNLM-PVDF was fabricated by filtration coating from a ZN suspension prepared by fractionation and redispersion of the thermally activated ZNs.

#### 2.5.1. ZNLM-Nafion Fabrication

After the UV activation, the entire multilayered ZN film detached from the hydrophobic PVDF (d_p_~0.22 μm) because of the lack of bonding between the polymer and ZN surface and shrinkage of the activated ZN layer. Recast Nafion films made in-house from the Nafion^®^ solution were used for fabrication of ZNLM membranes in a Nafion/ZNLM/Nafion sandwich structure. The Nafion film casting process was the same as that reported in reference [12]. First, the 20 wt.% Nafion solution was mixed with methanol, ethanol, and N,N-dimethylformamide at room temperature under stirring. The volumetric ratio of the components of the cast solution were 1.25 (20% Nafion solution); 0.75 (methanol, 99.8%); 0.85 (ethanol, 99.5%); and 3.0 (DMF, 99.8%). A total of ~24 mL of this final solution was placed into a Teflon petri dish (diameter of 3 inches) and dried by evaporating the solvents in a vacuum oven at 80 °C for 20 h under a pressure of 23.7 kPa. The dried film was further treated for 4 h at 120 °C under the same vacuum pressure. This resulted in a 110 μm thick dry Nafion film that was then brushed with the 20 wt. % Nafion solution on one side. The fully wetted surface was immediately pressed on the UV-activated ZN film loosely covered on the PVDF film. The ZN film was entirely attached to the Nafion film by pressing with a flat Teflon plate. The free side of the ZNLM surface was subsequently brushed with the 20 wt.% Nafion solution to strengthen the adhesion and form an ~10 μm thick Nafion film on the top. The ZN film sandwiched by the Nafion layers, i.e., the ZNLM-Nafion, was then dried at 40 °C in air followed by curing at 120 °C for 4 h under a vacuum pressure of ~23.7 kPa.

#### 2.5.2. ZNLM-PVDF Fabrication

The ZNLM on the porous PVDF film was coated by vacuum filtration of a controlled amount of ZN suspension following a procedure described in our previous publication [5]. The suspension for membrane coating contained a minimal amount of PVDF (0.06 wt.%) dissolved in the DMSO/EtOH (1:2 ratio) mixed solvent, which was used as binder to physically attach and retain the ZNLM film on the porous PVDF substrate (d_p_~0.45 μm).

The key to formation of a uniform and pinhole-free thin ZNLM is the high-degree dispersion state of the thermally activated ZSM-5 ZNs. To achieve this, the calcined ZNs were sonicated in 0.1 M KOH + 1.0 M KCl (pH~13) solution and 0.01 M HCl + 1.0 KCl solutions (pH~2–3) alternately for one week. The ZNs were filtered and redispersed in a water + EtOH mixture with a water/EtOH weight ratio of 80/20. The suspension was stirred overnight and peptized with 1 M HNO_3_ solution at pH of 3–4. Sedimentation and centrifugation were used to remove the aggregated ZNs. The fast-settling large ZN agglomerates in the bottom portion and the smallest debris remaining in the top section of the liquid column were removed. The remaining suspension contained relatively uniform and large-sized ZNs, which was ~10% of the initially retrieved ZNs. Therefore, the overall yield of open-pore ZNs by the thermal activation method was only ~1% from the initially synthesized rhombus crystals. The dispersion and aggregate removal were repeated until particle settling was not observed over 30 min under static condition. It should be noted that the ZN dispersibility did not change appreciably when suspension pH was reduced from ~7, where the ZN zeta potential (ζ) was −49.57 ± 1.79 mV, to 3–4, where ζ was −12.05 ± 1.81 mV.

These well-dispersed ZNs were recovered by a syringe filter and redispersed into EtOH. This suspension of ZNs in EtOH was then reformulated into the composition for coating ZNLM on the porous PVDF by vacuum filtration [5]. The final ZN suspension for membrane coating contained 0.02 wt.% ZN + 0.06 wt.% dissolved PVDF in an EtOH/DMSO solution mixed at an EtOH: DMSO weight ratio of 2:1. The continuous ZN-laminated layer was obtained by total filtration of 3 mL suspension over the 9.1 cm^2^ PVDF surface. The resultant PVDF-supported ZNLM, i.e., ZNLM-PVDF, was dried at 80 °C for 3 h under vacuum pressure of ~1.5 kPa followed by a 3 h curing process at 120 °C under an absolute pressure of ~23.7 kPa.

### 2.6. Proton Conduction in Aqueous Solutions

The ZSM-5 ZNLMs supported on the ionic Nafion films and nonionic macropore PVDF substrate were investigated for ion diffusion and proton-selective ion conduction in aqueous electrolyte solutions containing proton and vanadyl cations (VO^2+^) that are relevant to the aqueous all-vanadium redox flow battery (VRFB).

*Proton/vanadyl ion diffusion.* The measurements of ion permeation rates were performed by a procedure described in our previous work [9,12]. The membrane was mounted in a Teflon permeation cell and the solutions on the two sides were continuously circulated for effectively mixing solutions in the two compartments and minimizing concentration polarization at the membrane surfaces. The membrane was fully soaked with DI water when mounted before circulating the solutions to start the measurement. The feed side was circulated with 10 mL of 4/7 M VO^2+^ sulfate solution in 4/7 M H_2_SO_4_, and the permeate side was circulated with 20 mL of 1 M MgSO_4_ solution. The 1 M MgSO_4_ solution was used in the permeate side to balance the osmotic pressure and ionic strength between the two sides. The pH value and VO^2+^ concentration in the MgSO_4_ solution were monitored as a function of time using a pH meter (Thermo Scientific Orion 320) and a UV/vis spectrometer, respectively.

The ion fluxes $J_{i}$ (i= H^+^ and VO^4+^; mol/cm^2^‧h) were estimated from the time-dependency of their concentrations ($C_{p,i}$) in the permeate side tank in the linear region, i.e., the slope $(dC_{p,i}/dt$) when $C_{p,i}$ was far smaller than the feed concentrations ($C_{f,i}$),

(2) $$J_{i}=\frac{V_{p}}{A_{m}}\cdot \frac{dC_{p,i}}{dt}$$

where the balancing solution volume in the permeate side was $V_{p}$= 20 mL. The permeation selectivity $\alpha_{H/V}$ is defined as the ratio of proton flux ($J_{H^{+}}$) to vanadyl ion flux ($J_{V^{4+}}$), and the actual transport selectivity ($\alpha \prime_{H/V}$) can be obtained through normalization of the $\alpha_{H/V}$ by the ratio of driving force, which is ($C_{f,H^{+}}/C_{f,V^{4+}}$) when $C_{p,i}\ll C_{f,i}$,

(3) $$\alpha_{H/V}=\frac{J_{H^{+}}}{J_{V^{4+}}}\;\mathrm{and}\;\alpha \prime_{H/V}=\alpha_{H/V}/\left(C_{f,H^{+}}/C_{f,V^{4+}}\right)$$

*Proton conduction.* The proton conduction of the membrane was evaluated through the measurement of area specific electric resistance (ASR) by electrochemical impedance spectroscopy (EIS). The measurement was performed by a cell of typical flow battery structure where the membrane was sandwiched between two compressed carbon felt electrodes to form a membrane-electrode assembly (MEA). The active area of the mounted membrane was 2.0 cm^2^. This MEA was then held between two dense graphite discs of which the outer surfaces were attached to two copper sheet terminals by silver adhesive. The complete apparatus was described in our previous reports, which included the single cell, a Reference-600™ Potentiostat (Gamry Instruments Inc., Warminster, PA, USA), and two electrolyte tanks under circulation by tube pumps [9,12]. The EIS measurements were conducted when both sides of the membrane were circulated with a 2 M H_2_SO_4_ solution.

## 3. Results and Discussion

### 3.1. The As-Synthesized ZNs

**Figure 2:**
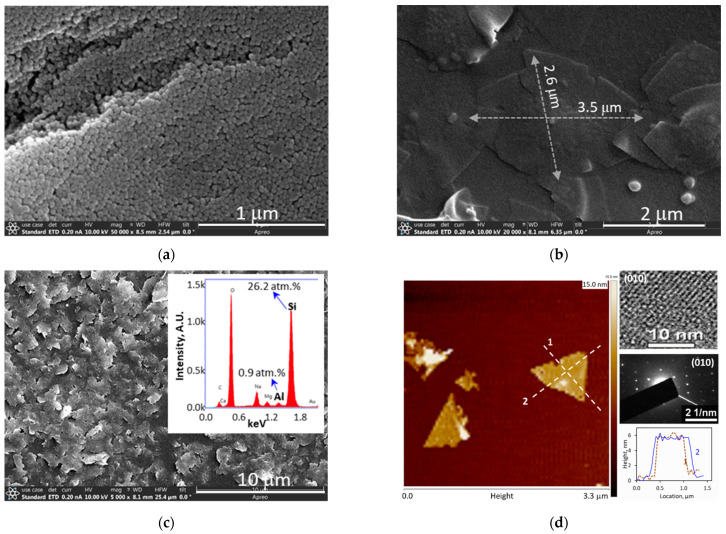
SEM images of (**a**) silicalite nanoparticle seeds [13], (**b**) as-synthesized ZMS-5 crystals by secondary growth using dC_5_ SDA, (**c**) nonactivated ZSM-5 ZN segregated after sonicated disintegration (insert showing an Si/Al ratio of ~29 ± 3 by EDS survey), and (**d**) AFM imaging height profile with TEM and electron diffraction pattern confirming ZN thickness orientation in *b*-axis direction.

Figure 2a,b show the SEM images of the silicalite nanoparticle seeds, which were ~30 nm in diameters, and the ZSM-5 crystals grown from the seeds, which were in typical rhombus shapes with diagonal dimensions of ~2.6 μm × 3.5 μm on average [6,7]. The flat ZNs obtained by removing the debris from the thick cores were in irregular shapes with average sizes around 1.0 μm × 1.2 μm and an overall framework Si/Al atomic ratio of 29 ± 3 (Figure 2c). The AFM height survey and TEM imaging and electron diffraction examinations in Figure 2d revealed that the ZNs were approximately 6.0 nm thick with the *b*-axis near-cylindrical straight channels (5.3 Å × 5.6 Å, Figure 1c) oriented along the thickness. The thickness was slightly smaller than what was found in our previous work [7] likely because of the more extensive washing by the aggressive alkaline solution that more thoroughly removed surface residuals.

### 3.2. Activation and Redispersion of ZNs

**Figure 3:**
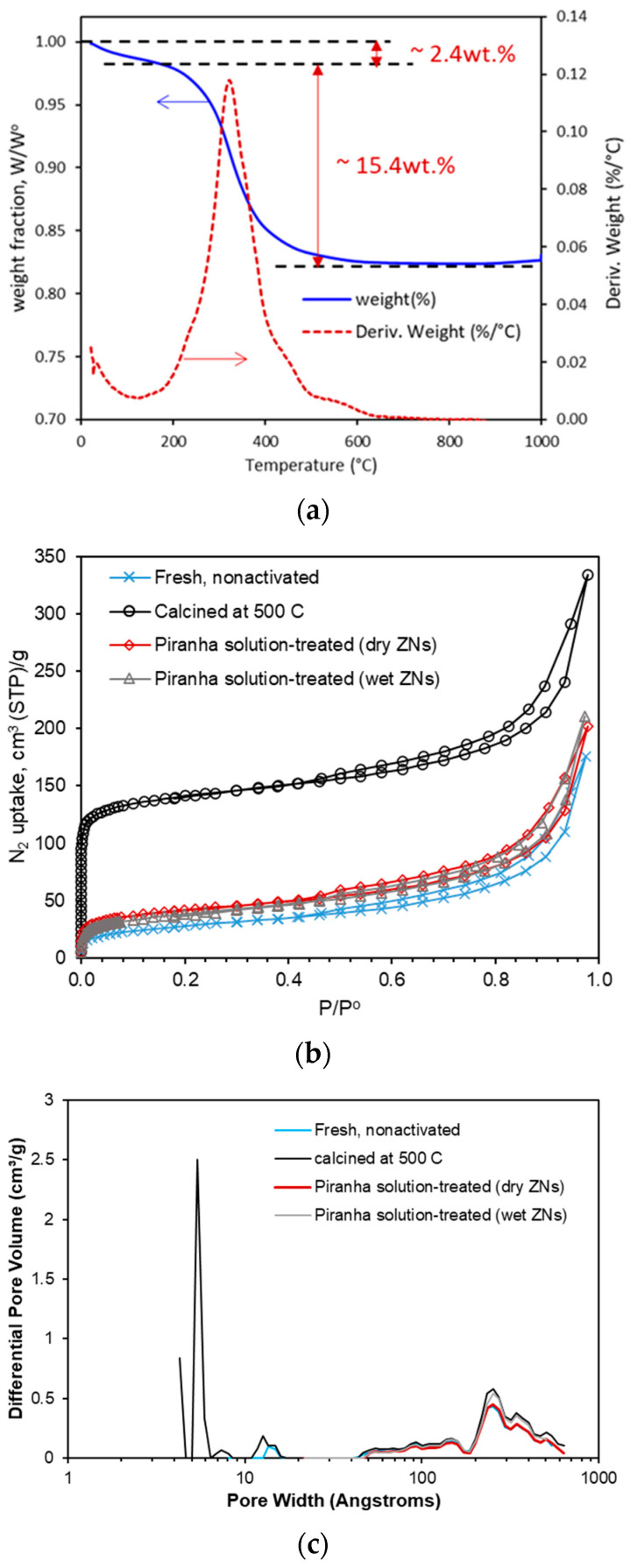

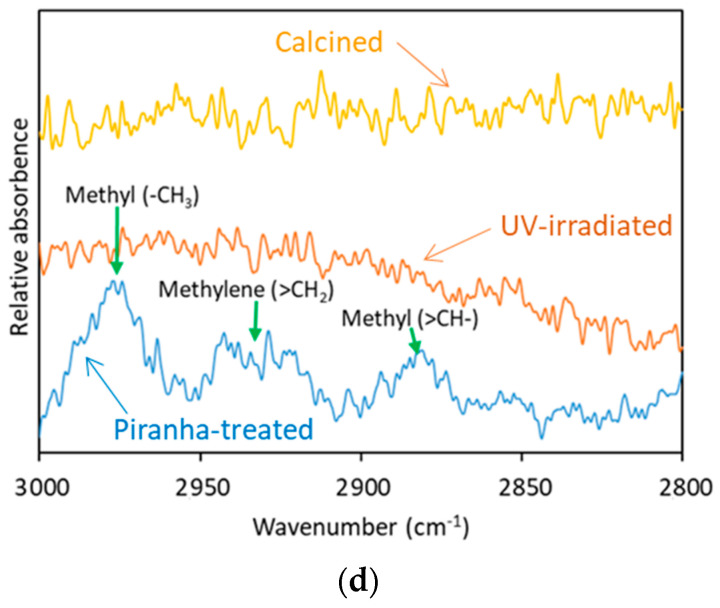
Characterizations for ZN samples after different treatments: (**a**) results of TGA measurement in air, (**b**) 77 K N_2_ adsorption–desorption isotherms for the fresh (nonactivated), calcined, and piranha solution-treated samples, (**c**) pore size distributions from the 77 K N_2_ isotherms in (**b**), and (**d**) the ATR−FTIR spectra (2800–3000 cm^−1^) of the calcined, UV−activated (3−day irradiation), and piranha solution-treated samples.

Figure 3a presents the TGA results, which verified the complete removal of dC_5_ from the ZSM-5 ZNs by the calcination method. The first weight loss (~2.4 wt.%) at up to 140 °C was caused by removal of externally adsorbed water and organics, and the second weight loss (~15.4 wt.%) at around 325 °C was caused by the thermal decomposition and removal of dC_5_ from the zeolitic pores. The N_2_ adsorption and desorption isotherms for the ZN samples are presented in Figure 3b. Although the piranha solution treatment was reported to be successful in decomposing the long-chain C_22-6-6_ SDA in the 3.2 nm thick (i.e., dimension of ~1.5 cells) silicalite ZNs [4], it was found to be inefficient for dC_5_ removal from the ~6 nm thick (i.e., length of ~3-unit cells) ZSM-5 ZNs.

As shown in Table 1, the BET surface area was 518 ± 17 m^2^/g for the calcined ZNs, which is typical for fully activated MFI ZNs [14,15]. The BET surface areas were <150 m^2^/g for the piranha solution-treated samples, which were only slightly greater than the nonactivated sample (97 m^2^/g). The conventional MFI crystals had a BET surface area of 392 ± 15 m^2^/g. The pore size distributions in Figure 3c showed micropores of characteristic MFI channel diameters of d_p_~0.56 ± 0.2 nm for the calcined samples, which were nonexistent for the fresh and piranha solution-treated samples. These indicate that the calcined ZNs had the dC_5_ completely removed while the piranha treatment was inefficient for the ZNs activation. The ineffective activation of ZSM-5 ZNs by piranha solution could be attributed to the higher stability of the short-chain dC_5_ molecules as well as the larger thickness (3-unit cells) and ionic surface of the ZNs that could hinder the access of zeolitic pores to oxidizing agents such as H_2_O_2_.

The amount of ZN suspension used in each batch of UV activation was <1 mg, which was far less than the amount of 50 mg required for the BET test. Thus, the FTIR spectroscopic method was used to investigate the removal of SDA from the ZSM-5 ZNs by UV irradiation via comparison with the calcined and piranha solution-treated samples. The ATR-FTIR spectra in Figure 3d revealed that organic SDA remained in the ZNs after the piranha solution treatments as indicated by the peaks of $-CH_{3}$, $=CH_{2}$ and =CH−. These characteristic peaks of the organic groups were absent in the sample calcined at 500 °C and in the sample treated with 3 days of UV irradiation. The ZNs for ZNLM fabrication were activated by 6 days of UV irradiation to ensure complete SDA removal. The removal of dC_5_ was further verified by the absence of carbon in the 3-day and 6-day UV-activated films deposited on an alumina disc according to the EDS examinations (Table 2).

The ZN film for calcination activation was also loaded on the alumina disc. Thus, the Si/Al ratios in Table 2 do not represent the composition of the zeolite frameworks because of the support influence. The Si/Al ratios of the ZSM-5 ZNs were around 30 based on the EDS measurements for the polymer-supported films (Figure 2c). The untreated fresh ZN film had a carbon-to-silicon (C/Si) atomic ratio of 0.31. The C/Si ratio was reduced to ~0.044 after 3 days, and further reduced to ~0.006 after 6 days of UV irradiation. The sample calcined at 550 °C for 8 h, which could be considered as a reference of total SDA removal, had a C/Si ratio of ~0.002. Although the EDS measurement has large error, the comparison of C/Si ratios strongly evidenced complete SDA removal after the 6-day UV irradiation. Thus, the ZN film treated with 6 days of UV irradiation was used for constructing the ZNLM-Nafion.

**Figure 4:**
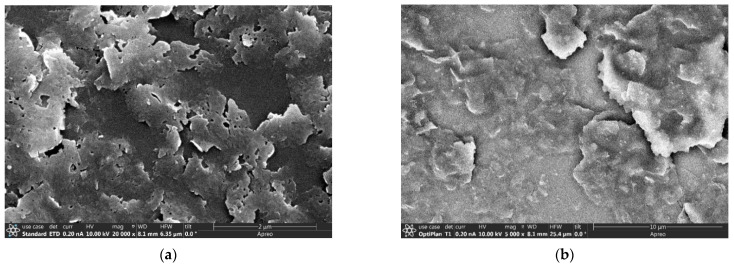
SEM pictures of the ZN deposits (**a**) from the suspension (0.02 wt.%; pH~3) of redispersed calcined ZNs and (**b**) from the suspension (0.02 wt.%; pH~3) of redispersed UV-activated ZN films.

*Redispersion of the activated ZNs.* The ZNs activated by calcination and the ZN films activated by UV irradiation were tested for redispersion in water by stirring and sonication in acidic and basic solutions as described in Section 2.3 and Section 2.5. Highly dispersed suspensions were obtained from the thermally activated ZNs, which could be seen from the absence of agglomerate texture in the film deposited from the suspension (Figure 4a). However, only a very small portion (~1.0%) of the initially made ZNs was utilized in the final suspension. The same dispersion procedure was unable to break up the large aggregates in the UV-activated ZN films even after additional treatments with alternated stirring and sonication for 24 h (Figure 4b). The tightly aggregated ZNs were presumably bound through water condensation of the silanol groups (i.e., ≡Si-OH + HO-Si≡ → ≡Si-O-Si≡ + H_2_O) between the contacting ZN surfaces under UV irradiation in dry conditions. These chemically bonded ZN piles are not easily dissociated and redispersed into individually separated ZNs.

Apparently, the chemically bonded irreversible aggregation was much less between ZNs in the calcined sample than in the UV-activated multilayered ZN films. For calcination, the dry ZNs were loosely and randomly spread out, which resulted in a low degree of direct contact between ZN surfaces. In contrast, the ZN film formed by vacuum filtration for UV activation was multilayered with ZNs that were well aligned to have maximized contact between the overlapping ZN surfaces. Therefore, the UV-activated ZN film could be used in a single piece to form the sandwich-structured ZNLM-Nafion membrane. The thermally activated ZNs were used for membrane synthesis on the porous PVDF by vacuum filtration of the dilute suspension containing 0.02 wt.% ZNs and 0.06 wt.% dissolved PVDF binder. The amounts of the ZNs per unit area were controlled in order to be the same in both Nafion and PVDF-supported ZNLMs for comparisons in ion diffusion and proton conduction tests.

### 3.3. ZNLM Membranes

**Figure 5:**
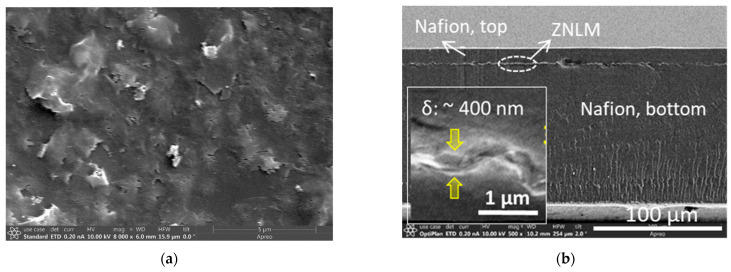

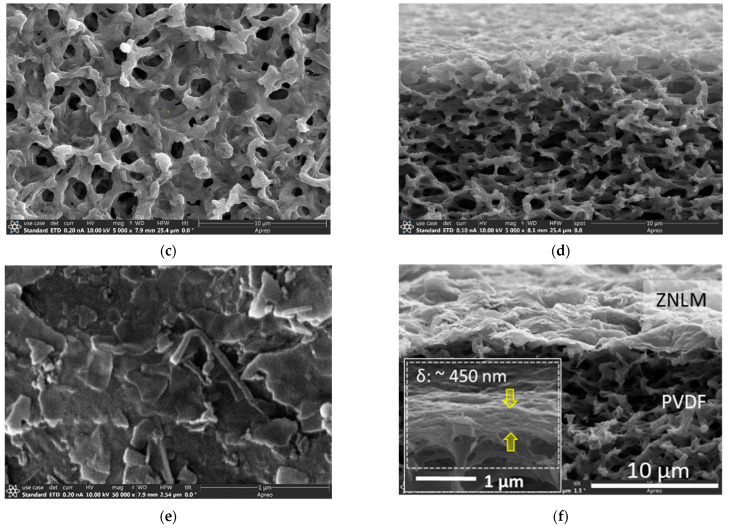
SEM images of the substrate and ZNLMs: (**a**,**b**) surface of the UV-activated ZN film on the base Nafion film before casting the top Nafion layer and cross-section of the entire Nafion-sandwiched ZNLM, respectively; (**c**,**d**) surface and cross-section of the bare PVDF substrate, respectively; and (**e**,**f**) surface and cross-section of the ZNLM-PVDF, respectively.

Figure 5 shows the SEM images of the ZNLM-Nafion and ZNLM-PVDF. The surface of ZNLM (Figure 5a) transferred onto the smooth Nafion film was uniformly laminated and free of pinholes before the Nafion overcoating (Figure 5b). The PVDF film had a rough surface because of the large pore size, which appeared to be much bigger than the manufacturer-stated 0.45 μm (Figure 5c,d). The ZNLM surface on PVDF was hilly but with no cracks (Figure 3e) because the ZN layer conformed to the rough surface of PVDF (Figure 5f). The microscopic images in Figure 5a,f also revealed that the ZNs were horizontally laid on the substrate surfaces; thus, the straight channels along the *b*-axis in the ZNs were oriented in the membrane thickness direction.

Accurate determination of the membrane thickness was difficult because the cross-section of ZNLM layers on the polymers tended to deform when deeply dried and frozen in liquid nitrogen for sample preparation. Nevertheless, thicknesses of ~400 nm and ~450 nm could be roughly estimated for the ZNLMs on Nafion (insert in Figure 5b) and PVDF (insert in Figure 5f), respectively. Based on the silicalite density ($\rho_{z\;}$= 1.76 g/cm^3^) [16] and the 6.0 × 10^−4^ g/cm^2^ ZNs deposited on the substrate, a thickness of ~375 nm could be expected if the ZN layer were free of inter-ZN spaces, i.e., as a single crystal film. The ZNLMs were apparently thicker because of the inter-ZN spaces in the multilayer structure. The inter-ZN spaces were expected to be larger in the ZNLM on PVDF than in the ZNLM on Nafion because the former contained binding PVDF chains between ZNs while the latter was binder free with ZNs interconnected by “≡Si-O-Si≡” bonds.

### 3.4. Proton Transport and Conduction

**Figure 6:**
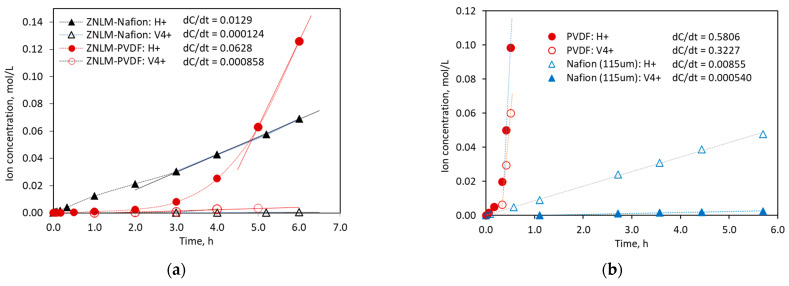
Ion concentrations, i.e., $C_{p,H^{+}}$ and $C_{p,V^{4+}}$, in the permeate tanks as a function of diffusion time for (**a**) the ZNLM-Nafion and ZNLM-PVDF and (**b**) the bare PVDF and recast Nafion membrane [13,17].

Figure 6 presents the results of ion diffusion measurements for the ZNLMs and the recast Nafion and porous PVDF substrates. Both the ZNLM-Nafion and ZNLM-PVDF (Figure 6a) exhibited markedly increased $\alpha_{H/V}$ accompanied by drastic reductions of ion fluxes ($J_{i}$) compared with their respective bare substrates (Figure 6b). The values of $J_{i}$, $\alpha_{H/V}$, and $\alpha \prime_{H/V}$ are listed in Table 3.

Because the ZNs in the ZNLM layer on Nafion support are bonded by ≡Si−O−Si≡ between overlapping ZN surfaces, the $\delta_{v}$ in the ZNLM on Nafion was expected to be much smaller than that on the PVDF as evidenced by the smaller thickness of the former. In addition, due to the existence of flexible PVDF chains between the ZNs in the ZNLM on PVDF, the $\delta_{v}$ could be further increased when the ZN surfaces were ionized and solvated in the strongly acidic electrolyte solutions, while the layered ZNs on the Nafion were tightly bonded to prevent opening of the inter-ZN spaces in the solution. In Figure 6a, the ZNLM-PVDF exhibited a slow increase in $C_{p,H^{+}}$ with time in the initial ~2 h and then transitioned into a much faster and steadily increasing period after 3–4 h. The initial slow increase in $C_{p,H^{+}}$ likely resulted from the displacement of presoaked water, the inter-ZN space evolution by surface protonation/solvation, and development of permeation through mixed paths of intra-ZN and inter-ZN porosities. In contrast, as can be seen in Figure 6a, the ZNLM-Nafion had a slightly smaller $C_{p,H^{+}}$ increasing rate in the beginning because the fully hydrated sulfonated polytetrafluoroethylene surface could instantly release H^+^ into the pH-neutral MgSO_4_ solution. The small variation of the $C_{p,H^{+}}$ increasing rate on the ZNLM-Nafion may be attributed to the slight delay by the ultrathin ZNLM in the middle. Apparently, without inter-ZN space evolution in the solution, the ultrathin ZNLM layer on the Nafion allowed rapid proton permeation with minimal resistance. As a result, the ZNLM-Nafion exhibited a moderately higher $\alpha_{H/V}$ of 104 and a lower $J_{H^{+}}$ of 1.29 mol/m^2^‧h compared with the ZNLM-PVDF, which had an $\alpha_{H/V}$ and $J_{H^{+}}$ of 73.2 and 6.28 mol/m^2^‧h, respectively.

The differences of $J_{H^{+}}$ and $\alpha_{H/V}$ between ZNLM-PVDF and ZNLM-Nafion could also be partially attributed to the different transport properties of the substrates. The PVDF was of a nonselective nature with low resistance while the Nafion film had a relatively high resistance to ion transport but good proton selectivity by itself. For the bare PVDF substrate, both $C_{p,H^{+}}$ and $C_{p,V^{4+}}$ increased rapidly after a relatively slow rate in the first ~0.3 h (Figure 6b), which was caused by the gradual displacement of the presoaked water and stabilization of the transmembrane concentration gradients. The $J_{H^{+}}$ and $J_{V^{4+}}$ through the bare PVDF were 58.1 mol/m^2^‧h and 32.3 mol/m^2^‧h, respectively, which gave an $\alpha_{H/V}$ of 1.8 and $\alpha \prime_{H/V}$ of 0.9. Thus, the porous PVDF alone was nonselective to H^+^ (H_3_O^+^ in solution) and VO^2+^. The recast 115 mm thick Nafion membrane was used to represent Nafion layers sandwiching the ZNLM because the amounts of sulfonated fluoropolymer were about the same. Unlike the nonionic PVDF, the Nafion membrane had no appreciable delay of proton permeation (Figure 6b) because of its strongly acidic surface. The $J_{H^{+}}$ and $J_{V^{4+}}$ for the 115 mm thick Nafion membrane were 0.855 mol/m^2^‧h and 0.054 mol/m^2^‧h, respectively, with an $\alpha_{H/V}$ of 15.8 and an $\alpha \prime_{H/V}$ of 7.9. The PVDF, after filling with the acidic electrolyte, had much higher $J_{H^{+}}$ with no H+ selectivity ($\alpha \prime_{H/V}$ near 1) mainly because its pore size (d_p_~0.45 μm) was much larger than the nanometer-scale water channels in the hydrated Nafion film (d_p_~2.5 nm [18]).

**Figure 7:**
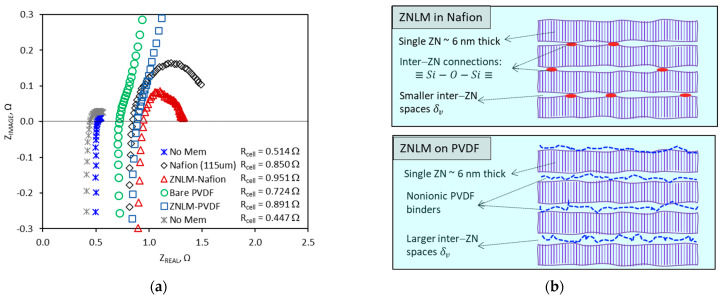
(**a**) Results of EIS measurements for the ZNLM–Nafion, ZNLM–PVDF, bare PVDF, and recast Nafion membrane, and (**b**) Schematics showing the ZN layered structures.

The ZNLM-Nafion and ZNLM-PVDF were tested for proton conduction in comparison with the bare substrates. Figure 7a presents the EIS Nyquist plots measured for the membranes and supports. The ASR values listed in Table 3 were determined based on the equivalent electric circuit of the test cell and the serial resistor model for the multilayered membranes described in our previous publications [9,19]. The ASR of the ZNLM-PVDF (~0.590 $\Omega \cdot cm^{2}$) was lower than that of the ZNLM-Nafion (~0.677 $\Omega \cdot cm^{2}$) primarily because the ASR of the macroporous PVDF (~0.350 $\Omega \cdot cm^{2}$), when filled with 2 M H_2_SO_4_, was significantly lower than that of the recast Nafion support (~0.531 $\Omega \cdot cm^{2}$). The low ASR of the ZNLM membrane was apparently a result of its thinness along which the straight channels in the *b*-axis were oriented preferably for ion transport. However, the ZNLM layer on the PVDF substrate appeared to be electrically more resistive (ASR~0.240 $\Omega \cdot cm^{2}$) than the ZNLM film sandwiched between the Nafion films (ASR~0.146 $\Omega \cdot cm^{2}$). Although the number of ZN layers were presumably the same in both ZNLM layers, the UV-activated ZNLM layer on Nafion was less resistant because of the minimized inter-ZN width $\delta_{v}$ by direct (≡Si−O−Si≡) connections and the highly acidic ZSM-5 ZN surfaces (${\left[AlO_{2}\right]}^{-}H^{+}$) (Figure 7b). On the other hand, the ZNLM on the PVDF exhibited larger ASR most likely because the nonionic PVDF binding chains obstructed ion transport through the inter-ZN spaces (Figure 7b).

**Figure 8:**
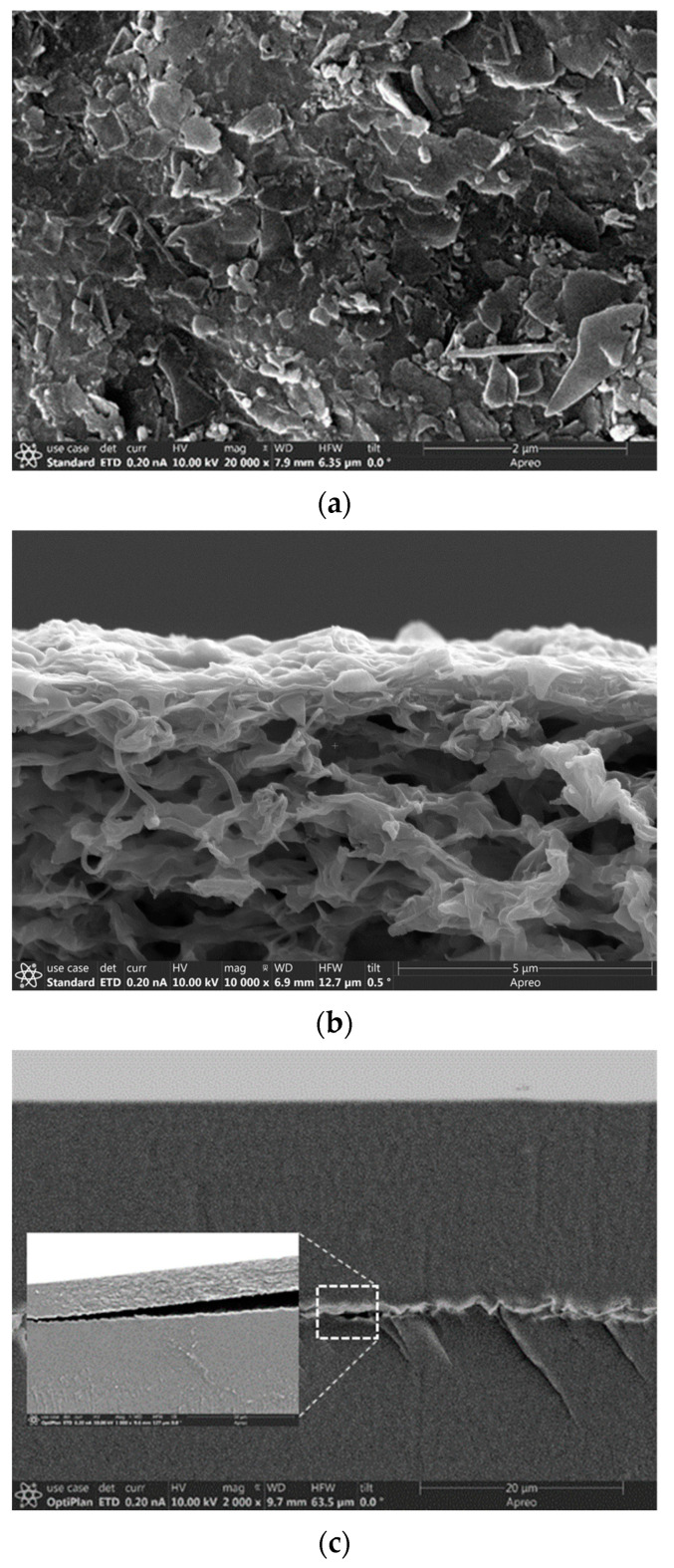
The SEM images of the supported ZNLM membranes after ~2 weeks of operation in the concentrated acidic electrolyte solutions: (**a**,**b**) ZNLM-PVDF surface and cross-section, respectively, and (**c**) cross-section revealing detachment between the ZNLM and Nafion layers.

The SEM examination was conducted for the ZNLM membranes after operating in the 2 M H_2_SO_4_ and acidic vanadyl ion solution for over two weeks. The SEM images of ZNLM-PVDF showed no visible changes in the surface morphology and multilayered structure (Figure 8a,b). However, SEM images revealed locations in the ZNLM-Nafion where the ZNLM and Nafion layers detached (Figure 8c). The structural integrity of the ZNLM-PVDF was retained by the physically tied multilayered ZNs, and the entire ZNLM layer was strongly adhered to the substrate of the same PVDF polymer as the binder. However, in the ZNLM-Nafion, because the ZSM-5 ZNs and Nafion films both had anionic frameworks and extra-framework proton compensators, the interfacial contact dissociated upon solvation of the surface protons in aqueous solutions.

## 4. Conclusions

This study demonstrated the ability of multilayered ZSM-5 zeolite nanosheet (ZN) films for highly selectivity proton conductions in aqueous electrolyte solution with low resistances. The ZSM-5 ZN-laminated membrane (ZNLM), formed on the nonionic macropore PVDF substrates using the same PVDF as a binder, was able to achieve a comparably low ASR but much higher proton selectivity compared with the Nafion membrane of similar thickness. This indicates the potential for development of ZNLMs as efficient proton-conducting membranes without using expensive ionic polymer matrices. However, the development of such new ZN-laminated membranes on ubiquitous polymer is limited by the inefficiency in preparing well-dispersed suspensions of open-pore ZNs, which are necessary for coating membranes on polymer supports. Our current research efforts are focused on developing methodologies for synthesizing dispersible open-pore ZNs and investigating the ZNLM performance as an ion separator for aqueous flow batteries.

## Figures and Tables

**Table 1:** BET surface areas measured by 77 K N_2_ adsorption–desorption for the ZSM-5 ZNs after calcination and piranha solution treatments.

Sample	BET Surface Area, m^2^/g
As synthesized (nonactivated)	97 ± 11
After calcination at 500 °C	518 ± 17
After piranha solution treatment (dry powders) *	147 ± 13
After piranha solution treatment (wet powders) **	134 ± 10
Conventional MFI crystal (calcined at 500 °C)	392 ± 15

* The dry ZNs were dispersed into the piranha solution; ** the wet ZNs filtered from suspension were added into the piranha solution without drying.

**Table 2:** The EDS elemental analyses for ZSM-5 ZNs before and after UV irradiation and calcination at 550 °C.

Elements	Before Activation	UV Irradiation for 3 days	UV Irradiation for 6 days	Calcined at 550 °C for 6 h
C	5.7 ± 1.5	~0.8	~0.1	~0.03
O	59.4 ± 0.4	54.9 ± 0.7	53.6 ± 0.4	54.2 ± 0.4
Al	9.1 ± 2.5	18.5 ± 2.0	22.6 ± 0.8	22.5 ± 3.5
Si	18.4 ± 1.3	18.2 ± 2.0	16.1 ± 1.2	15.8 ± 2.8
C/Si ratio	~0.310	~0.044	~0.006	~0.002

**Table 3:** The $J_{H^{+}}$, $J_{V^{4+}}$, $\alpha_{H/V}$, $\alpha \prime_{H/V}$, and ASR of the ZNLM membranes and bare substrates.

Membrane	δ, µm	$J_{H^{+}}$ mol/m^2^·h	$J_{V^{4+}}$ mol/m^2^·h	$\alpha_{H/V}$	$\alpha \prime_{H/V}$	*ASR*, Ω‧cm^2^
ZNLM-Nafion	~110	1.29	0.0124	104.0	52.0	0.677
ZNLM-PVDF	~125	6.28	0.0858	73.2	36.6	0.590
Recast Nafion [12]	~115	0.855	0.054	15.8	7.9	0.531
PVDF support [13]	~125	58.1	32.3	1.8	0.9	0.350
ZNLM on Nafion	~0.40	--	--	--	--	0.146
ZNLM on PVDF	~0.45	--	--	--	--	0.240

## Data Availability

Data are contained within the article.
